# The temporal evolution of a facial pain syndrome associated with neurovascular contact: a case report

**DOI:** 10.1186/s10194-015-0497-5

**Published:** 2015-02-11

**Authors:** Sabrina Khan, Ida Wibrandt, Per Rochat, Messoud Ashina

**Affiliations:** Danish Headache Center, Department of Neurology, Glostrup Hospital, Faculty of Health and Medical Sciences, University of Copenhagen, Nordre Ringvej 57, Glostrup, DK-2600 Denmark; Department of Neurosurgery, Copenhagen University Hospital, Rigshospitalet, Denmark

**Keywords:** Trigeminal neuralgia, Trigeminal autonomic cephalalgias, Cranial autonomic symptoms, Neurovascular contact, Microvascular decompression

## Abstract

**Background:**

Trigeminal autonomic cephalalgias are primary headaches characterized by unilateral pain and cranial autonomic symptoms. However, associated autonomic symptoms have also been reported in other headaches and facial pains, e.g. trigeminal neuralgia, with the clinical differentiation proving a complex task.

**Case:**

A 54-year-old man presented with right-sided, sharp, intense facial pain in the distribution area of the trigeminal nerve. Pain duration was from seconds to a few minutes, and trigger factors included ipsilateral touching of the skin and hair. Over the next ten years, symptoms progressed and changed presentation, also displaying as right-sided, severe, orbital pain, lasting 60 to 90 minutes, with conjunctival injection and rhinorrhea. Neurological examination was normal. Numerous medications were tried with limited or no effect. In 2010, magnetic resonance imaging revealed a right-sided deviation of the basilar artery at the level of pons, creating neurovascular contact with the trigeminal nerve. Microvascular decompression was performed, and symptoms resolved within days.

**Conclusion:**

Differentiating between trigeminal autonomic cephalalgias and trigeminal neuralgia with autonomic symptoms can be challenging. The distinct change and evolution over time in the clinical presentation of the patient’s head pain suggests a temporal plasticity of the pain in head and facial syndromes, irrespective of underlying pathoanatomic features.

## Background

Trigeminal autonomic cephalalgias (TACs) comprise a debilitating group of primary headaches characterized by unilaterality of pain and pronounced ipsilateral cranial autonomic symptoms (CAS), such as conjunctival tearing, lacrimation, and nasal congestion [1]. TACs include cluster headache (CH); short-lasting unilateral neuralgiform headache attacks with conjunctival injection and tearing (SUNCT), paroxysmal hemicrania, and hemicrania continua. The main distinction between TACs is based on pain duration and differences in abortive and preventive treatments. Trigeminal neuralgia (TN) is characterized by unilateral, brief, electric shock-like pains, abrupt in onset and termination, limited to the distribution of one or more divisions of the trigeminal nerve (TG). Pain is triggered by innocuous stimuli, e.g. tooth brushing or chewing [1]. While CAS are distinguishing features of TACs, autonomic symptoms have also been reported in association with TN, proving it difficult to differentiate between these two head pains [2,3].

We present the case of a patient with unilateral head pain attacks and prominent CAS who underwent a remarkable progression and evolution in the clinical presentation of his head pain, and ultimately responded completely to microvascular decompression (MVD) of the TG.

## Case presentation

A 54-year-old male with history of obesity, type II diabetes, and medically treated hypertension, presented in year 2000 with facial pain. One month prior to presentation, he was involved in a car accident with no obvious trauma to the neck. He suffered from neck pain shortly after, but was never seen by a physician. At presentation, the patient described a sensation of sharp, unilateral pain in the right-sided sensory distribution area of TG, lasting from 1 second to 3 minutes (Table 1). Attack frequency was 4–5 per day, in intervals of 15 minutes to 4 hours. Triggers included ipsilateral touching of the skin or hair. This pattern continued for up to 3 weeks, interrupted by 14 days of pain remission.

**Table 1:** **Summary of symptom development**

**Time**	**Pain localization & characteristics**	**Triggers**	**Pain duration**	**Attack frequency**	**Cluster duration**	**Associated CAS**	**Presumed diagnosis**
**Onset**	Right-sided, sensory area of TG, sharp, intense.	Right-sided touching of hair or skin	1 s – 3 min in intervals of 15 min – 4 hrs.	4-5 x daily	1-3 weeks	No	TN
**+2 yrs.**	Unchanged	Increased	Unchanged	Increased		Unchanged	Atypical facial pain
**+9 yrs.**	Right-sided, supraorbital, radiating towards teeth & ear	Right-sided speaking, eating, tooth-brushing	5-10 min	5-6 x daily	Constant	No	TN
	Right eye, intense, sharp	None	60-90 min	5-6 x daily	1 month	Yes	CH
**+10 yrs.**	Right eye, radiating to teeth, stabbing, electric shocks	Unchanged	10-15 min	25 x daily	Constant	Yes	TN with CAS

Neurological examination was normal, apart from reported hypoesthesia in the right-sided V2. TN was suspected and the patient was started on oxcarbazepine 900 mg daily. This initially eliminated the pain but caused cognitive side effects. Gabapentin was then tried, but discontinued due to skin exanthema. Two years after onset, the patient experienced an acute exacerbation of the facial pain with an increase in attack intensity and frequency. Indomethacin treatment was initiated, which successfully reduced the intensity but not the attack frequency. A private practicing neurologist diagnosed the patient with atypical facial pain. Over the next 2 years, the symptoms progressed further. There were fewer remitting periods, the pain intensity grew, and trigger factors now also included tooth brushing, chewing, shaving, exposure to windy weather and drinking cold beverages. Several antiepileptic drugs were tried without significant effect on the symptoms.

Magnetic resonance (MR) imaging and MR angiography performed 4 years after onset showed a loop on the basilar artery at the level of pons. Due to a possible correlation between neurovascular contact and presentation of neuralgia, MVD surgery was suggested, but the patient declined.

9 years after onset the patient was referred to the Danish Headache Center and Department of Neurology, Glostrup Hospital, Copenhagen. At this time he presented with two different facial pains. The first pain was right-sided, supraorbital, radiating towards the ipsilateral ear and teeth, lasting 5 to 10 minutes, with a frequency of 5–6 per day. Trigger factors included speaking, eating and tooth brushing; there were no associated autonomic symptoms. The second pain was sharp and intense, in the right eye, followed by conjunctival injection and rhinorrhea, lasting 60 to 90 minutes with a frequency of 5–6 attacks per day. Based on past and present history the patient was diagnosed with both TN and CH, and started on verapamil 480 mg daily later increased to 720 mg. This treatment continued for a year, reducing the symptoms greatly. Oxygen was also tried, but with no substantial effect.

10 years after onset the patient was hospitalized due to further worsening of the condition, with rapidly increasing attack frequency. He complained of episodes of intense facial pain, described as constant, stabbing, electrical shocks around the right eye, radiating towards the ipsilateral teeth, and associated with prominent ipsilateral autonomic features including conjunctival injection, lacrimation, periorbital edema, miosis, ptosis, rhinorrhea and facial redness. Attacks occurred as paroxysms of pain that lasted 10 to 15 minutes, with a frequency of 25 attacks per day. Trigger factors persisted as earlier. The condition was interpreted as TN with autonomic symptoms and the patient was tried on phenytoin loading, with immediate effect. Daily treatment with phenytoin 400 mg and carbamazepine 800 mg was initiated. After a few days the patient reported symptom relapse. He was then tried on indomethacin 200 mg daily, with no effect.

**Figure 1:**
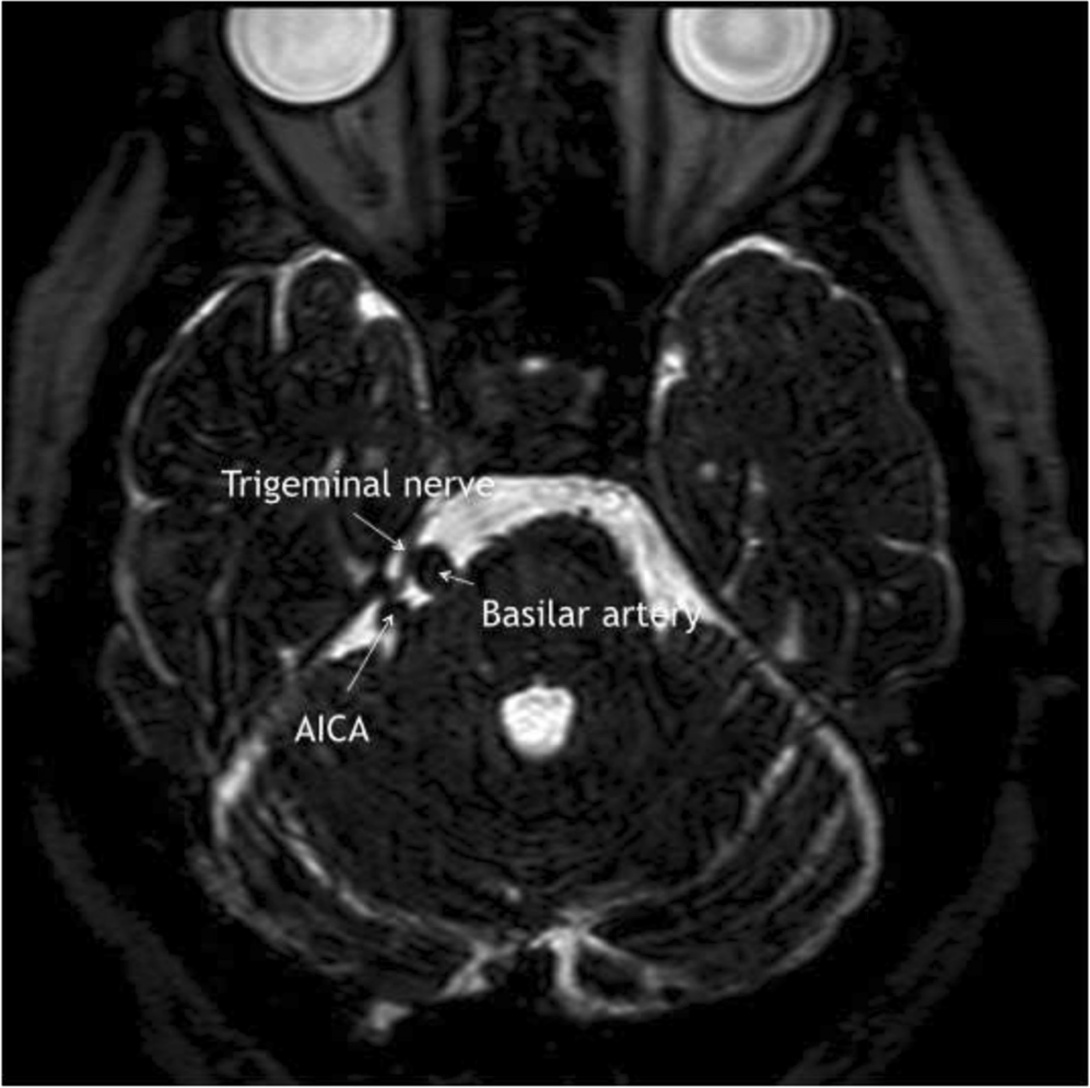
**MRI showing right-sided neurovascular contact between the basilar artery and the trigeminal nerve.** While it may look like the anterior inferior cerebellar artery (AICA) is in fact also compressing the trigeminal nerve, the treating neurosurgeon (PR) did not find this to be the case during surgery.

A new MRI confirmed a loop on the basilar artery, dislocating the TG in lateral direction, resulting in neurovascular contact between artery and nerve (Figure 1). The patient was immediately referred to surgery and underwent MVD. Upon surgery, the neurosurgeon reported as follows: “The trigeminal nerve is located behind a large petrosal sinus, which is coagulated and divided. This provides overview of the trigeminal nerve, where the basilar artery is seen to indent the anterior side of the nerve. A small branch of the basilar artery is seen to depart from behind the trigeminal nerve, however without causing any direct neurovascular contact. The nerve is separated from the artery, and a piece of felt/Teflon is placed in between”.

At clinical controls performed 2 weeks, 3 months, 10 months, 2 and 4 years post surgery, the patient reported complete pain remission, with no need for prophylactic therapy. His quality of life had increased significantly.

## Conclusions

Over the course of ten years, the patient experienced a prominent change in the phenotype of his head pain, with severe progression in pain intensity, duration, and associated CAS. The question remains whether the facial pains were associated or in fact presentations of distinct conditions. At first glance, the pain characteristics fit TN and CH, respectively. TN attacks last from seconds to 2 minutes. Treatment options include carbamazepine and gabapentin, which had some effect on the patient but was discontinued due to adverse events. However, the presence of autonomic symptoms may disturb this proposed diagnosis, as CAS are only sparingly described in relation to TN without co-existence of SUNCT [4,5]. Over time the attack duration progressed in the patient, and while this may initially point towards SUNCT rather than TN, a new study has shown that concomitant persistent pain is prevalent in TN and may represent a distinct phenotype of this pain syndrome [6]. Reporting on 158 TN patients, Maarbjerg and colleagues also reported that in patients with concomitant persistent pain, autonomic symptoms prevail in 37% and V1 involvement in 31%.

CH is characterized by unilateral, orbital or supraorbital pain, with associated autonomic symptoms, lasting 15 to 180 minutes. Attacks occur in clusters of weeks or months, with subsequent remission periods of months or years. However, while verapamil reduced the patient’s symptoms, oxygen provided no relief. Also, CH is not associated with a trigger area on the face. MVD had full effect on the patient, and while MVD in CH is not as well-defined as in TN, a study of 39 chronic CH patients undergoing MVD of the TG alone or in combination with decompression or sectioning of the nervus intermedius, showed initial post-operative success of at least 50% pain relief achieved in 73.3% of patients. However, long-term follow-up revealed a drop in this success rate to 46.6% [7].

Differentiating between TN with V1 autonomic symptoms and TACs, namely SUNCT, can prove tricky [3,4,8]. Ophthalmic distribution, prominent autonomic symptoms, longer pain duration, lack of refractory period and relative lack of response to carbamazepine are described as differing features [2]. While trigger factors are typical of TN, several authors have concluded that SUNCT may in fact also be subject to precipitating mechanisms [3,4,9]. In a study of 21 SUNCT patients, Pareja and Sjaastad showed that although paroxysms in SUNCT are located within the V1 distribution area, triggering areas may also be located within V2 and V3, and even include extratrigeminal areas, such as the neck [9]. Several articles have discussed the presence of autonomic symptoms in TN and a possible difference between their presentation in TN and SUNCT [3,4]. Pareja et al. conclude that the autonomic component of TN is milder than in SUNCT and for TN also may appear later in the attack [3]. It has also been proposed that attacks of V1 TN of long duration may overlap the shortest SUNCT attacks, thereby comprising a continuum of symptomatology [10], and begging the question whether these syndromes are in fact interchangeable and possibly subsets of one another.

While we acknowledge the possibility of retrospective recall in this case with symptom development over 10 years, it is important to note that a physician saw the patient regularly and at every change of the headache phenotype. Neurologists with knowledge of headache symptomatology, presentation, and treatment performed the headache description, and we believe that this close follow-up by relevant physicians reduces the risk of retrospective bias.

Despite initial confusion, the immediate and sustained effect of MVD proposes that the different pain presentations most likely were rooted in one, sole condition. While TN is well known to develop due to neurovascular contact and MVD is standard treatment [11,12], MVD is also reported to be effective in various TACs, where neurovascular contact or vascular malformations may exist as underlying pathology [7,13-15].

The truly compelling aspect of this case is the prominent change over time in the patient’s clinical presentation, leaving the involved physicians perplexed as to the true etiology of the head pain. While raising the question whether TN with V1 autonomic symptoms may progress to presentations of TACs, this case suggests that pain is not necessarily a static measure, but may change and evolve over time, while maintaining its underlying pathoanatomic features.

To our knowledge, a similar and equally distinct variation in the phenotype of a facial pain has not been reported earlier. Longitudinal follow-up within this field may provide a better comprehension of the true temporal evolution and possible plasticity of facial pain syndromes, all in the effort of optimized and timelier treatment.

## Consent

The patient provided oral consent to the corresponding author for publication of this case report and any accompanying images.
